# Supplementary material for: Causal Relationship between Aging and Anorexia Nervosa: A White-Matter-Microstructure-Mediated Mendelian Randomization Analysis
Source: Biomedicines. 2024 Aug 16;12(8):1874. doi: 10.3390/biomedicines12081874 (PMC11351342; doi:10.3390/biomedicines12081874)
Supplement: Supplementary file 1 [file biomedicines-12-01874-s001.zip › Supplementary Figure/Figure S4/Singe SNP plot of MR analysis for TL to ubm-b-1869.pdf]

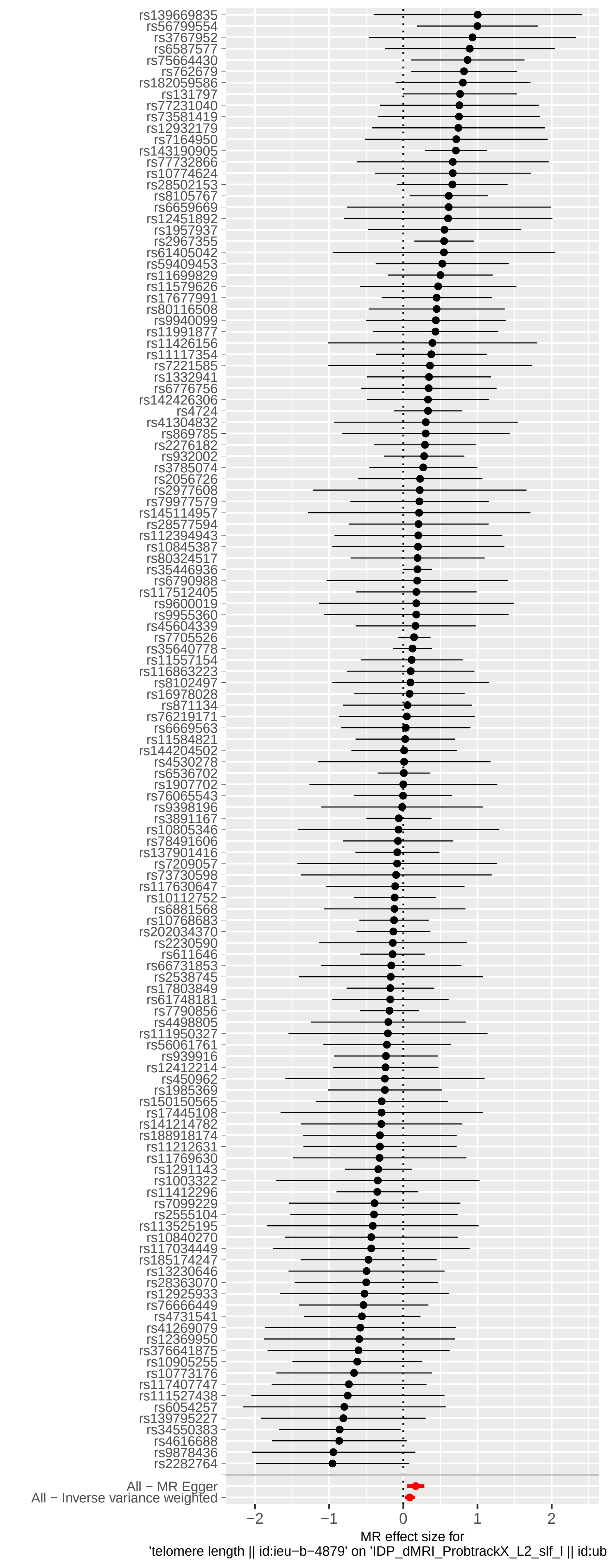

rs139669835
rs56799554
rs3767952
rs6587577
rs75664430
rs762679
rs182059586
rs131797
rs77231040
rs73581419
rs12932179
rs7164950
rs143190905
rs77732866
rs10774624
rs28502153
rs8105767
rs6659669
rs12451892
rs1957937
rs2967355
rs61405042
rs59409453
rs11699829
rs11579626
rs17677991
rs80116508
rs9940099
rs11991877
rs11426156
rs11117354
rs7221585
rs1332941
rs6776756
rs142426306
rs4724
rs41304832
rs869785
rs2276182
rs932002
rs3785074
rs2056726
rs2977608
rs79977579
rs145114957
rs28577594
rs112394943
rs10845387
rs80324517
rs35446936
rs6790988
rs117512405
rs9600019
rs9955360
rs45604339
rs7705526
rs35640778
rs11557154
rs116863223
rs8102497
rs16978028
rs871134
rs76219171
rs6669563
rs11584821
rs144204502
rs4530278
rs6536702
rs1907702
rs76065543
rs9398196
rs3891167
rs10805346
rs78491606
rs137901416
rs7209057
rs73730598
rs117630647
rs10112752
rs6881568
rs10768683
rs202034370
rs2230590
rs611646
rs66731853
rs2538745
rs17803849
rs61748181
rs7790856
rs4498805
rs111950327
rs56061761
rs939916
rs12412214
rs450962
rs1985369
rs150150565
rs17445108
rs141214782
rs188918174
rs11212631
rs11769630
rs1291143
rs1003322
rs11412296
rs7099229
rs2555104
rs113525195
rs10840270
rs117034449
rs185174247
rs13230646
rs28363070
rs12925933
rs76666449
rs4731541
rs41269079
rs12369950
rs376641875
rs10905255
rs10773176
rs117407747
rs111527438
rs6054257
rs139795227
rs34550383
rs4616688
rs9878436
rs2282764
All − MR Egger
All − Inverse variance weighted
−2
−1
0
1
2
MR effect size for
'telomere length || id:ieu−b−4879' on 'IDP_dMRI_ProbtrackX_L2_slf_l || id:ub
